# The Anti-cancer Effects of *Capparis spinosa* Hydroalcoholic Extract

**Published:** 2019

**Authors:** Yasaman Moghadamnia, Seydeh Narges Mousavi Kani, Maryam Ghasemi-Kasman, Mohamad Taghi Kazemi Kani, Sohrab Kazemi

**Affiliations:** 1. Neuroscience Research Center, Health Research Institute, Babol University of Medical Sciences, Babol, Iran; 2. Department of Pharmacology, Faculty of Medicine, Babol University of Medical Sciences, Babol, Iran; 3. Cellular and Molecular Biology Research Center, Health Research Institute, Babol University of Medical Sciences, Babol, Iran

**Keywords:** Cancer, Capparis, High Pressure Liquid Chromatography, Quercetin

## Abstract

**Background:**

Recently, due to the steep increase in cancer lethality statistics, pharmaceutical societies seek approaches for designing drugs with higher efficiency and lower expenses. Plant-based drugs have therefore gained much attention, due to their abundance and ease of accessibility, and their higher effectiveness.

**Methods:**

Wild-grown caper [*Capparis spinosa* (*C. spinosa*)] was collected from northern Iran and next 100 *g* of the powder was added to 300 *ml* of a solvent (Ethanol 80), the solution was mixed for 72 *hr* and later filtered *via* Whatman filter papers. The solvent was taken out under vacuum conditions and extracts were then collected and stored in glass vials. The High Pressure Liquid Chromatography (HPLC) method was used to assay quercetin which consisted of the following specifications: C18 column, UV detector wavelength of 260 *nm*, mobile phase acetonitrile and water and flow rate of 1 *ml/min*. In this study, the anti-cancer effects of *C. spinosa* extract on HeLa, MCF7, Saos and Fibroblast cancer cell lines have been investigated.

**Results:**

The amount of quercetin was assessed by HPLC. The anti-tumor activity and the antioxidant level of hydroalcoholic extract of *C. spinosa* have been evaluated with MTT assay and FRAP technique, respectively. HPLC data showed quercetin form the major component of *C. spinosa* extract. In addition, FRAP data indicated that *C. spinosa* extract had high antioxidant activity and MTT assay indicated that *C. spinosa* extract effectively decreased the cancer cell lines.

**Conclusion:**

The quercetin in *C. spinosa* extract had significant anti-tumor effects and may be regarded as an ideal natural drug for cancer therapy.

## Introduction

The second leading cause of death in the world is cancer. Treatment procedures are complicated for each specific type of cancer 1. The pharmaceutical societies mostly aim for anti-cancer drugs with higher effectiveness, less toxicities and lower costs. However, despite many advancements achieved in cancer treatment strategies, drug-resistance has been reported in many cancer treatment trials. It has been shown by experimental studies 2 that some known plants have anti-cancer effects on various tissue cells, which is due to the variety of compounds containing in plant drugs, as opposed to the purely synthesized version. It then seems that introducing new drugs derived from plants could lead to more efficient approaches to cancer treatment.

Through recent years, much attention has been paid to plant-based drugs 3, mostly due to vast developments in organic chemistry, groundbreaking changes in meth ods of extraction and purification and more precise understanding of the plants’ natural compounds. Additionally, using plant sources as cheaper raw material could help the pharmaceutical industries develop better drugs with much lower costs 4.

Capparidaceae are a vast family of phanerogam gymnosperm dialypetalae plant species. The *Capparis spinosa (C. spinosa)* (CS) as a member of the Capparidaceae family, not only shows noticeable resistance to low water supplies and high temperatures, but can also adapt to as low a temperature as −8 °*C* 5.

**Figure 1.**
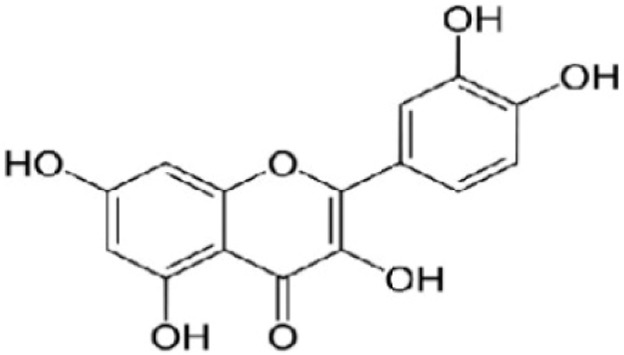
Structure of quercetin.

Flavonoids are known as the largest group of natural compounds that are strong antioxidants and have prominent effects in cellular biology, *e.g*. collecting free radicals and possibly preventing their damaging role in carcinogenesis 6,7. Research has shown that these compounds have an important effect in preventing genetic mutations that ultimately lead to generating cancerous tumors 8,9. *C. spinosa* contains an abundant amount of the flavonoids, more specifically a flavonoid compound called quercetin with molecular formula of C_15_H_10_O_7_ and molecular mass of 302.23 *g/mol* (Figure 1). This very compound has anti-inflammatory, anticoagulant, antibacterial, antihypertension and antiatherogenic properties 10,11. The results of another study indicated *C. spinosa* contains volatile and non-volatile compounds which play an important role in colon cancer prevention 12.

A number of previous studies have focused on the protective effects of flavonoids in liver diseases 13. It has been reported, as a result of the mentioned studies, that flavonoids help prevent hepatocellular carcinoma, reduce alcohol cirrhosis effects caused by oxidative stress, and slow down the growth rate of specific cancer cells *in vitro* 14.

It therefore seems, according to the antioxidant property of flavonoids and their effect on cancer cells, as well as an abundance of flavonoids in *C. spinosa*, that the hydroalcoholic extraction can reduce the growth and proliferation of cancer cells. In this study, the effects of CS extract on HeLa, MCF7 and Saos cancer cell lines proliferation were investigated.

## Materials and Methods

### Chemicals

Sodium carbonate, ascorbic acid, 1, 2, 4, and 6-Tris (1-pyridyl)-5-triazin (TPTZ) were purchased from Merck Chemicals (Darmstadt, Germany). Dulbecco’s Modified Eagle Medium (DMEM) and Fetal Calf Serum (FCS) were also prepared. Trypan Blue staining was also purchased from Invitrogen (Karlsruhe, Germany), and the 3-(4, 5-dimethylthiazol-2-yl)-2, 5-diphenyltetrazolium bromide (MTT) was from Sigma-Aldrich. All high purity High Pressure Liquid Chromatography (HPLC) grade solvents used for the analyses were from Daejung, Korea; except for formic acid which was obtained from Merck. Pure reference standard quercetin was purchased from Merck.

### Preparation of plant extracts and determination of yield

Wild-grown caper (*C. spinosa*) was collected from northern Iran, Firouzkouh heights, in June 2016. Initially, the aboveground organs of the plants were separated and dried under fume hood, afterwards grinded in to powder. Next, 100 *g* of the powder was added to 300 *ml* of a solvent (Ethanol 80). The solution was mixed for 72 *hr* using Labnet incubator shaker (made in USA), and later filtered *via* Whatman filter papers. Finally, the solvent was taken out under vacuum conditions using the rotary evaporator (from IKA, Germany) machine, therefore providing the ethanol extract. The extracts were then collected and stored in glass vials covered with aluminum foil and kept at 4 °*C* in refrigerator. The percentage of yielding extracts was calculated as follows:

$$\text{Yield}\;\text{percentage}=\frac{\text{weight}\;\text{of}\;\text{sample}\;\text{extract}}{\text{initial}\;\text{weight}\;\text{of}\;\text{sample}}\times 100$$

### Measuring quercetin level using HPLC

In order to measure the amount of quercetin in the resulting extract, 0.01 *g* of the extract was weighed and solved in 10 *ml* of the mobile phase solvent. Afterwards, it was filtered using HPLC 0.45 *μm* filters, injecting a final amount of 20 *μl* of the extract to the HPLC method.

### HPLC system and operating conditions

The HPLC method was used to assay quercetin which consisted of the following specifications: C18 column with 4.6 *mm* diameter and 25 *mm* length, UV detector wavelength of 260 *nm*, mobile phase acetonitrile and water with 80/20 ratio and flow rate of 1 *ml/min*.

### Cell culture

The Hela, MCF7, Saos and Fibroblast (Control group) cell lines were obtained from the Cell Bank of the Pasteur Institute of Iran. Then, these cells were kept in Cell Culture Laboratory in School of Public Health University of Medical Sciences and were cultured in RPMI 1640 medium (Gibco, USA) containing 5% fetal calf serum (Gibco, USA) and penicillin and streptomycin antibiotic mixture (Gibco, USA) in incubator at 37 °*C*, 5% CO_2_ pressure and saturated moisture. Cytotoxicity test was performed according to the previously published experiences. The MTT assay was repeated three times for each hand. Cells were cultured in 96-well plate for one night and were then treated with extract of caper for one night in incubator. The culture medium was then replaced with a new medium containing color solution. The cells were placed in the new medium for 3 *hr*, the supernatant was disposed and isopropanol was poured into the wells. Finally, the color of the medium was read at wavelength of 570 *nm* using ELISA reader.

### FRAP test

FRAP test was performed using TPTZ (2, 4, 6-tripyridyl-s-triazine). This method is based on revival of ferric iron to ferrous (Fe^3+^-TPTZ to Fe^2+^-TPTZ) in the presence of antioxidant. The FRAP solution contains 0.3 *M* acetate buffer (pH=3.6), 10 *mM* TPTZ in 40 *mM* HCI and 20 *mM* iron chloride solution (III) with ratios of 1-1-10. The FRAP solution must be prepared freshly. In this method, 100 *μl* diluted extract was mixed with 4.1 *ml* FRAP solution and the absorbance was read at a wavelength of 593 *nm* after 20 *min*.

### Statistical analysis

All the analyses were performed using one e way ANOVA followed by Tukey post-test when it needed Graph pad PRISM software. Data were determined to be significant when p<0.05.

## Results

**Figure 2.**
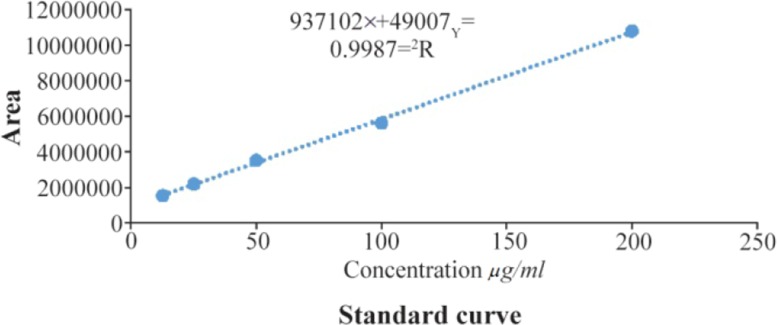
Standard curve of the quercetin.

In this research, extract containing quercetin was separated from *C. spinosa* by hydroalcoholic extraction. To determine the concentration of quercetin in CS extracted, standard curve was plotted with a minimum of 5 concentrations of quercetin in the standard solution (Figure 2). Standard peaks of quercetin at 12.5, 25, 50, 100 and 200 *μg/ml* were prepared.

**Figure 3.**
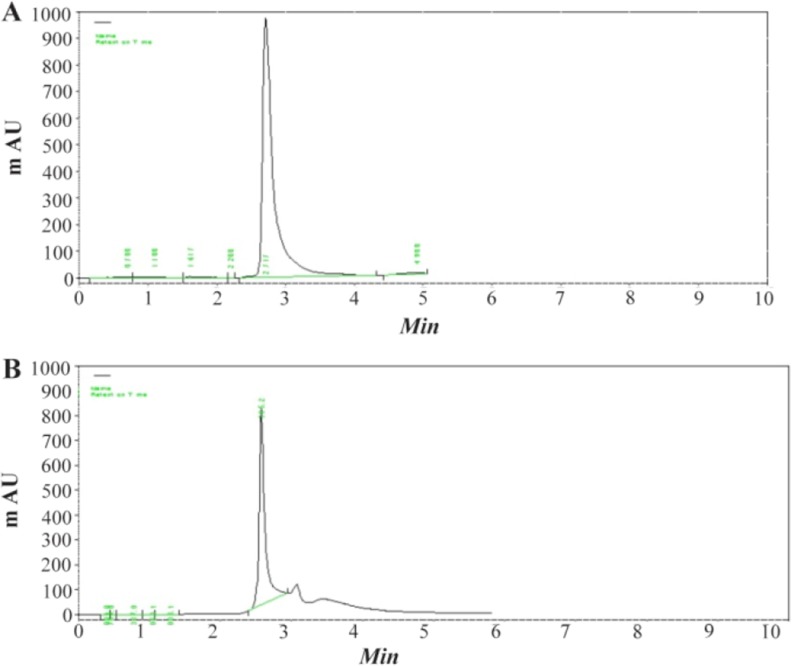
The sample peaks of quercetin, (2.8 *min*), A) standard quercetin (0.2 *mg/ml* and; B) *C. spinosa* sample (1 *mg/ml*).

Quantitative analysis of extracted quercetin was carried out using HPLC chromatograms of (a) quercetin standard and (b) after isolation of the extraction from aerial part and quercetin (10.06%) was identified as a major component (Figure 3).

The results from HPLC analysis has shown that CS is the main source of quercetin and could be used for extraction purposes and usage in drug manufacturing. Similar to our study, Germano MP *et al* conducted a study on CS and ultimately showed that the *C. spinosa* has a high nutritive value index (due to its high level of antioxidants). It was also suggested by their team that CS could be used as an agent in perfumes and as a flavoring in everyday use 15.

Due to detection of high levels of quercetin in CS (which is known to be a strong antioxidant) in FRAP and Hydroxyl Radical tests, it is inferred that this compound could be used as an anticancer material. The results of Al-Soqeer’s test of hot water extract of CS in rats has shown that it can have protective effects against lead acetate, which is due to a profusion of antioxidants in it 16. Natural polyphenols such as quercetin, galangin have long been used for the prevention and treatment of several disorders due to their antioxidant, cytotoxic, antineoplastic, and immunomodulatory effects 17,18.

Quercetin also has anti-inflammatory and wound healing effects. In a study done by Tajik *et al*, it was shown that different doses of the CS hydroalcoholic extract could have healing effects on tongue wounds in rats 19. Another study performed by Ozan *et al* on dental PDL cells have shown that this extract could be a fitting alternate for preservative media that are designed for holding avulsed teeth sound before performing replantation 20.

**Figure 4.**
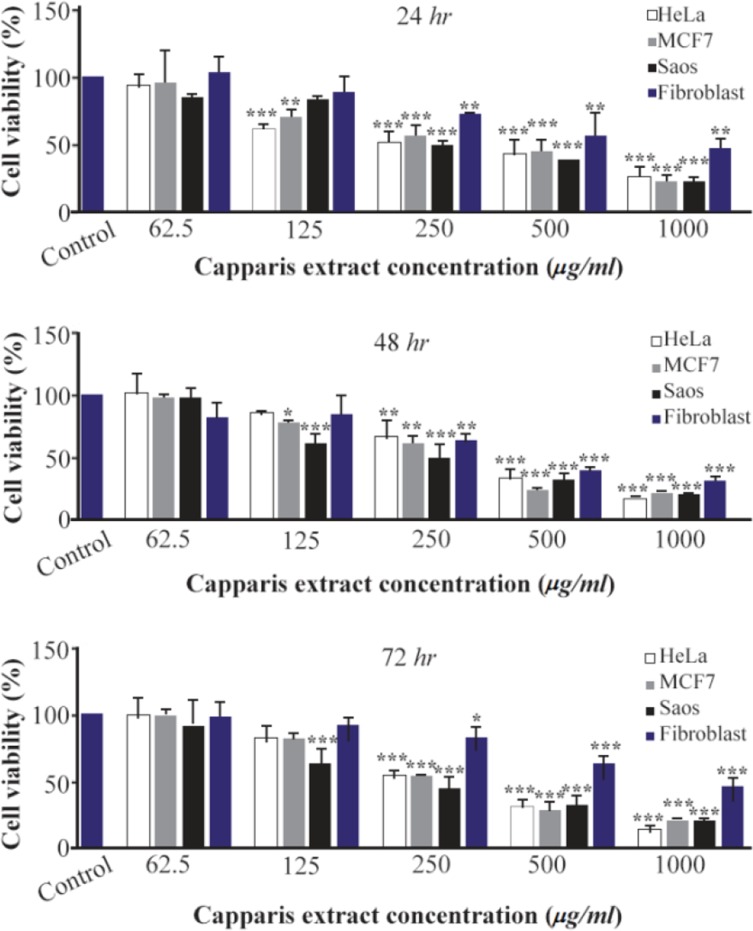
Cell viability percentage determined by MTT assay for (A) HeLa, (B) MCF7 (C) Saos and (D) Fibroblast cell lines after treatment with different concentrations of capparis extract.

**Figure 5.**
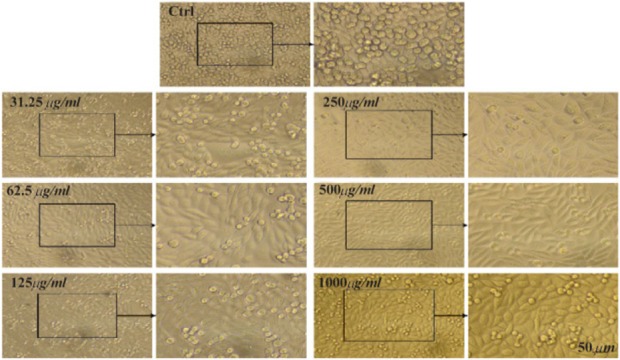
Microscopic images of Saos cell line after 72 *hr* of treatment with different concentrations of CS extract, scale bar: 50 *μm*.

**Figure 6.**
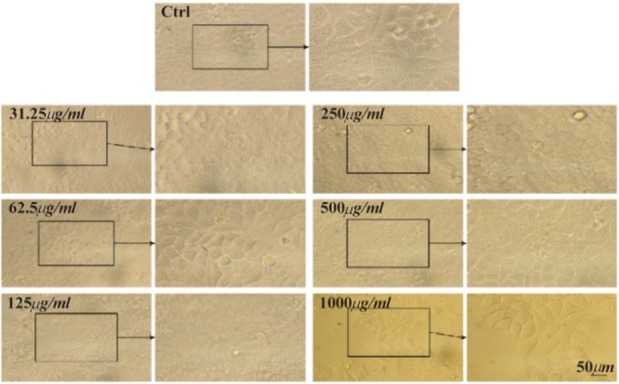
Microscopic images of Msf7 cell line after 72 *hr* of treatment with different concentrations of CS extract, scale bar: 50 *μm.*

**Figure 7.**
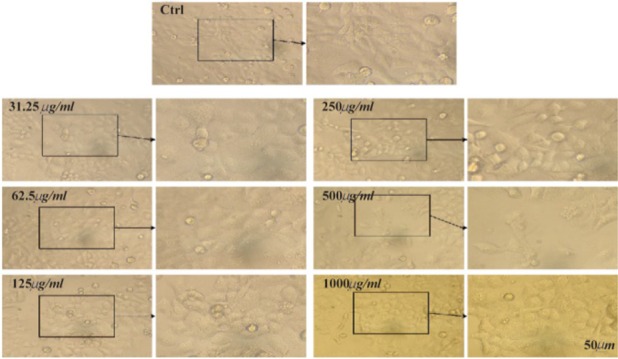
Microscopic images of HeLa cell lines after 72 *hr* of treatment with different concentrations of CS extract, scale bar: 50 *μm.*

The MTT assay test was performed on Hela, MCF7 and Saos cancer cell lines and the results are shown in figure 4. According to the results, the hydroalcoholic extract was toxic on three cell lines and it could be inferred that CS extract can prevent cancer cell growth in low concentrations 17. Figures 4–7, respectively, present the microscopic image of Hela, Saos and MCF7 cell lines with different concentrations of CS extract after 72 *hr*. According to figure 4, the best effective dose of drug for cancer cells compared to normal cells was 250 *μg/ml* after 72 *hr*. The high cell viability percentage of three cell lines shows that 1000, 500 and 250 *μg/ml* of CS extract had the minimum effect on healthy cells. Another similar study has been done by Kulisic-Bilusic *et al*, investigating the effects of CS extract on HT-29 cells, in which they stated that CS contains volatile and nonvolatile components that have potential effects in preventing colorectal cancer 12.

## Discussion

It has been shown that N-Butanol CS extract can induce apoptosis in SGC-7901 cells 21. Other comparable studies include the study of hydroalcoholic CS extract effects on Hela cells, done by Mansour RB *et al*. The results of this study show that the mentioned extract contains components like polyphenols and flavonoids, which can be used as a rich source of natural antioxidant molecules 22.

CS also contains many more healing properties, including its anti HIV-1 effects, anti-proliferative property especially in cancerous cells, antifungal effects of CS extract (mostly due to the proteins contained in it), healing effects in treating liver toxicity and oxidative stress caused as a result of 6-mercaptoporine. In addition to the mentioned properties, the hydroalcoholic CS extract can help with ossification through the primary stages 23–25.

## Conclusion

Multiple phytochemicals isolated from eatable plants have been reported to possess anticancer properties. Chemoprevention by eatable plants is now considered to be an inexpensive approach to cancer management. In the present study, it was shown that *C. spinosa* contains non-volatile compounds which potentially can play an important role in HeLa, MCF7 and Saos cancer prevention by inhibiting their respective tissues’ cancerous cells proliferation. Among other compounds, quercetin was detected as dominant. The anticancer activity of *C. spinosa* hydroalcoholic extract, as a good source of flavonoids, was reported in this study.
